# MitraClip step by step; how to simplify the procedure

**DOI:** 10.1007/s12471-016-0930-7

**Published:** 2016-12-08

**Authors:** M. A. Sherif, L. Paranskaya, S. Yuecel, S. Kische, O. Thiele, G. D’Ancona, A. Neuhausen-Abramkina, J. Ortak, H. Ince, A. Öner

**Affiliations:** 1 Cardiology Department, University Hospital Rostock, Rostock, Germany; 2 Cardiology Department, Vivantes Klinikum im Friedrichshain, Berlin, Germany; 3 Cardiology Department, Vivantes Klinikum Am Urban, Berlin, Germany

**Keywords:** MitraClip, Mitral regurgitation, Percutaneous mitral valve repair, Step by step

## Abstract

The MitraClip system is a device for percutaneous edge-to-edge reconstruction of the mitral valve in patients with severe mitral regurgitation who are deemed at high risk for surgery. Studies have underlined the therapeutic benefit of the MitraClip system for patients at extreme and high risk for mitral valve surgery, suffering from either degenerative or functional mitral regurgitation. The MitraClip procedure shows low peri-procedural complication rates, and a significant reduction in mitral regurgitation, as well as an improvement in functional capacity and most importantly quality of life. It hereby widens the spectrum of mitral valve repair for the Heart Team. The current review underscores the efficacy of the procedure and describes the technique to simplify the procedure.

## Introduction

The MitraClip (Abbott Laboratories, Menlo Park, California, USA) is a catheter-based technology that is similar to the Alfieri technique in that it connects the middle scallops of the anterior and the posterior leaflet of a regurgitant mitral valve. The data of the EVEREST (Endovascular Valve Edge-to-Edge Repair Study) trials [1, 2] and results of registries [3] demonstrate that the MitraClip procedure is feasible and safe. Procedural success has been shown to increase with operator experience [4].

A large prospective, nonrandomised European study, ACCESS-EU (the Amsterdam Center for Contemporary European Studies – A Two-Phase Observational Study of the MitraClip System in Europe) found 81.8% survival at 1 year and 78.9% freedom from severe mitral regurgitation (MR) [5]. Data from the GRASP (Getting Reduction of Mitral Insufficiency by Percutaneous Clip Implantation) registry at 30 days and 1 year showed promising results: freedom from the same composite endpoint of death, surgery, or severe MR in 75.8% of 117 treated patients at 1 year, and a 3.4% MAE rate at 30 days [6]. A meta-analysis reviewing 16 studies came to similar conclusions, documenting a low adverse event profile and only 14.7% of patients demonstrating severe MR at 1 year [7].

Several ongoing trials will help clarify the role of MitraClip therapy in the treatment of functional MR. The COAPT trial (Clinical Outcomes Assessment of MitraClip Percutaneous Therapy), a prospective, randomised, parallel controlled study, is currently enrolling patients with severe functional MR and heart failure (left ventricular ejection fraction 20–50%). A similar study, the RESHAPE-HF trial (Randomized Study of the MitraClip Device in Heart Failure Patients With Clinically Significant Functional Mitral Regurgitation), is enrolling patients with severe functional MR and a left ventricular ejection fraction of 15–40%, deemed non-surgical candidates who will be randomised to MitraClip therapy versus medical management.

In this review we describe the technical aspects of the MitraClip procedure.

## Pre-procedural planning

Planning for the clip procedure requires definition of the mitral valve morphology and the specific pathology causing regurgitation. The depth/distance to the coaptation point of valve leaflets is crucial to determine the appropriate clip-landing site, but valve pathophysiology dictates the ideal transseptal puncture point [8].

## Eligibility criteria

**Fig. 1:**
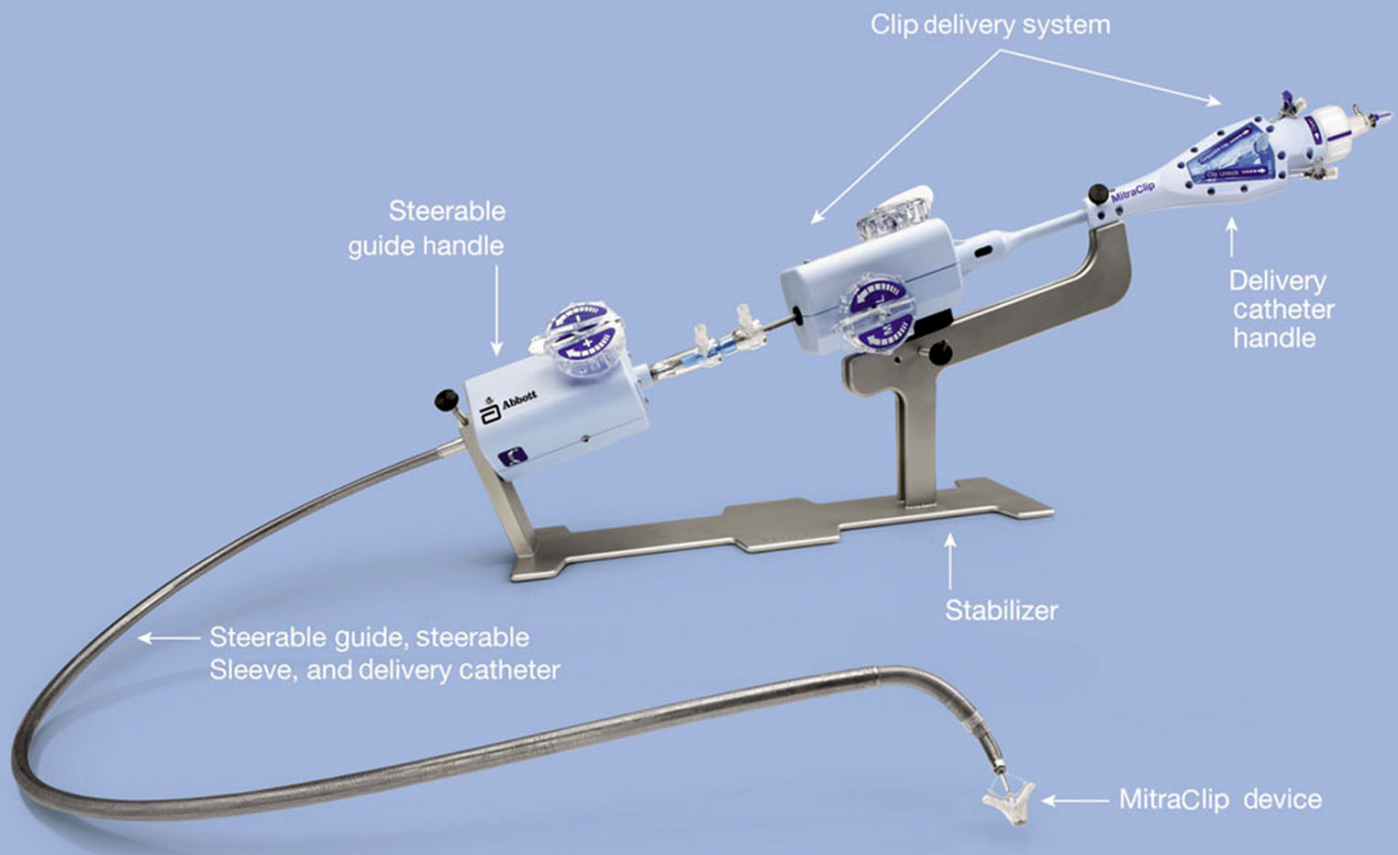
Components of the MitraClip system (Images provided and adapted from Abbott Laboratories, Menlo Park, California, USA)

To guarantee safe positioning of the clip, anatomical eligibility criteria are recommended. A coaptation length of >2 mm, a coaptation depth of <11 mm, and in the case of degenerative disease, a flail gap of <10 mm and a flail width of <15 mm are favourable [8]. The MitraClip system consists of a steerable guide catheter that is introduced using the transfemoral route, and by echocardiographic guiding, transseptally into the left atrium. The clip delivery system can be introduced through the guide catheter (Fig. 1).

## Determination of the morphology

The mitral valve morphology and the cause of MR should be assessed in detail by transoesophageal echocardiography (TEE), as suitable mitral valve morphology is essential to a successful MitraClip procedure. Transthoracic echocardiography is used to assess MR severity, but TEE is also useful to confirm the initial TTE findings [9].

Characterisation of valve pathology is important for pre-procedural planning. Degenerative valves causing significant MR often have substantial portions of the leaflet that are either flail or prolapsed. Patients with a flail or prolapse distance greater than 1 cm width along the line of coaptation or greater than 1 cm into the left atrium do not meet the criteria for clip procedure and are likely to have poor procedural success [10].

Assessment of the subvalvular apparatus and chordae is also important since the extended arms of the clip are easily entangled within these structures. Short chordae or unusual angles of chordae usually portend trouble and warrant careful avoidance when possible [10, 11].

## Integration of 2D/3D TEE and fluoroscopy for MitraClip procedure

Two- and three-dimensional (2D/3D) TEE is used for guidance during the MitraClip procedure. Three-dimensional TEE provides en face views of the mitral valve, thus facilitating the assessment of mitral valve morphology and pathology, which is important for patient selection. Delivery catheters, wires, devices, and target structures can be visualised in one single view and in relation to each other, thus optimising transseptal puncture, steering of the delivery catheter in a 3D space (left atrium) towards the mitral valve and good MitraClip positioning perpendicular to the line of coaptation in the middle segments of the mitral valve.

Biner et al. [10] demonstrated that the usage of 2D and 3D TEE in combination is associated with a remarkable 28% reduction in procedure times. Fluoroscopy provides additional helpful information on the positioning and distance for delivery catheter advancement and MitraClip opening, orientation, and the final release of the device on the leaflets, most notably when a second MitraClip is implanted.

The MitraClip procedure is divided into the following steps: 1) Transseptal puncture; 2) Advancement of the steerable guide catheter into the left atrium; 3) Advancement of the clip delivery system into the left atrium and positioning of the MitraClip below the mitral valve leaflets; and 4) Grasping of the leaflets, assessment of the result and clip release.

## Transseptal puncture

**Fig. 2:**
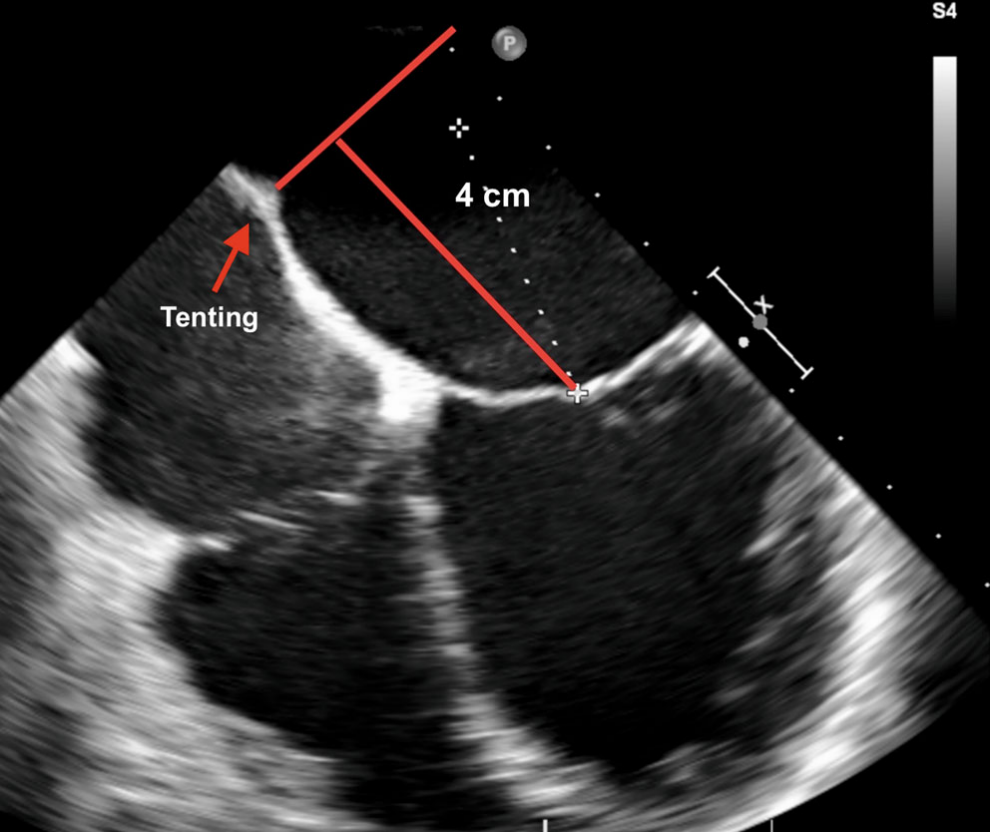
The puncture site is evaluated using TEE in a 4-chamber view, and the ‘tenting’ of the atrial septum can be seen as the transseptal needle is pushed against it, ideally in the superior and posterior part of the interatrial septum with the aim of obtaining adequate working space and distance above the mitral annulus

Transseptal puncture represents one of the most important aspects of the MitraClip procedure. The optimal puncture site is located superiorly and posteriorly in the interatrial septum and three TEE planes are used to determine the correct site: a short-axis view at the base for anterior-posterior orientation, a bi-caval view for superior (cranial)-caudal (inferior) orientation, and a four-chamber view to direct the height above the mitral valve. The position of the BRK transseptal needle (St. Jude Medical, Inc, St Paul, Minnesota, USA) can be seen by a tent-like indentation of the interatrial septum (‘tenting’). Thereby, the tip of the ‘tent’ points towards the left atrium. With a satisfactory posterior and superior location, the height above the valve is assessed in a four-chamber view (Fig. 2). The site of optimal transseptal puncture is different for degenerative and functional MR. In degenerative disease (e. g. prolapse), the puncture site needs to be 4–5 cm above the mitral annulus to guarantee enough space for adequate catheter and MitraClip manoeuvring. In contrast, in cases of functional MR, the line of coaptation is usually below the plane of the mitral annulus due to extensive tethering. Therefore, the puncture site in these patients needs to be more inferior and closer to the annular plane (about 3.5 cm above the annular plane).

## Introduction of steerable guide catheter into the left atrium

The steerable guide catheter with the dilator is gently advanced into the left atrium over an Amplatz extra stiff wire (placed in the left upper pulmonary vein) under fluoroscopic and TEE guidance. The dilator can be easily identified by a cone-shaped tip and a typical echogenic appearance of ridges on the catheter system. A radiopaque echo bright double ring characterises the tip of the guide catheter. The advancement of the steerable guide catheter should be carefully visualised under continuous 2D/3D TEE, and fluoroscopic monitoring to avoid injuries of the free left atrial wall. Once the catheter is safely placed in the left atrium, the Amplatz extra stiff wire is retrieved first, followed by the dilator.

## Advancement of the clip delivery system into the left atrium and positioning of the MitraClip below the mitral valve leaflets

The clip delivery system is advanced via the steerable guide catheter under fluoroscopic guidance directed toward the left upper pulmonary vein. TEE is additionally necessary to ensure that the tip of the steerable guide catheter remains across the interatrial septum and that the delivery system with the clip at the tip does not cause injury to the free left atrial wall.

**Fig. 3:**
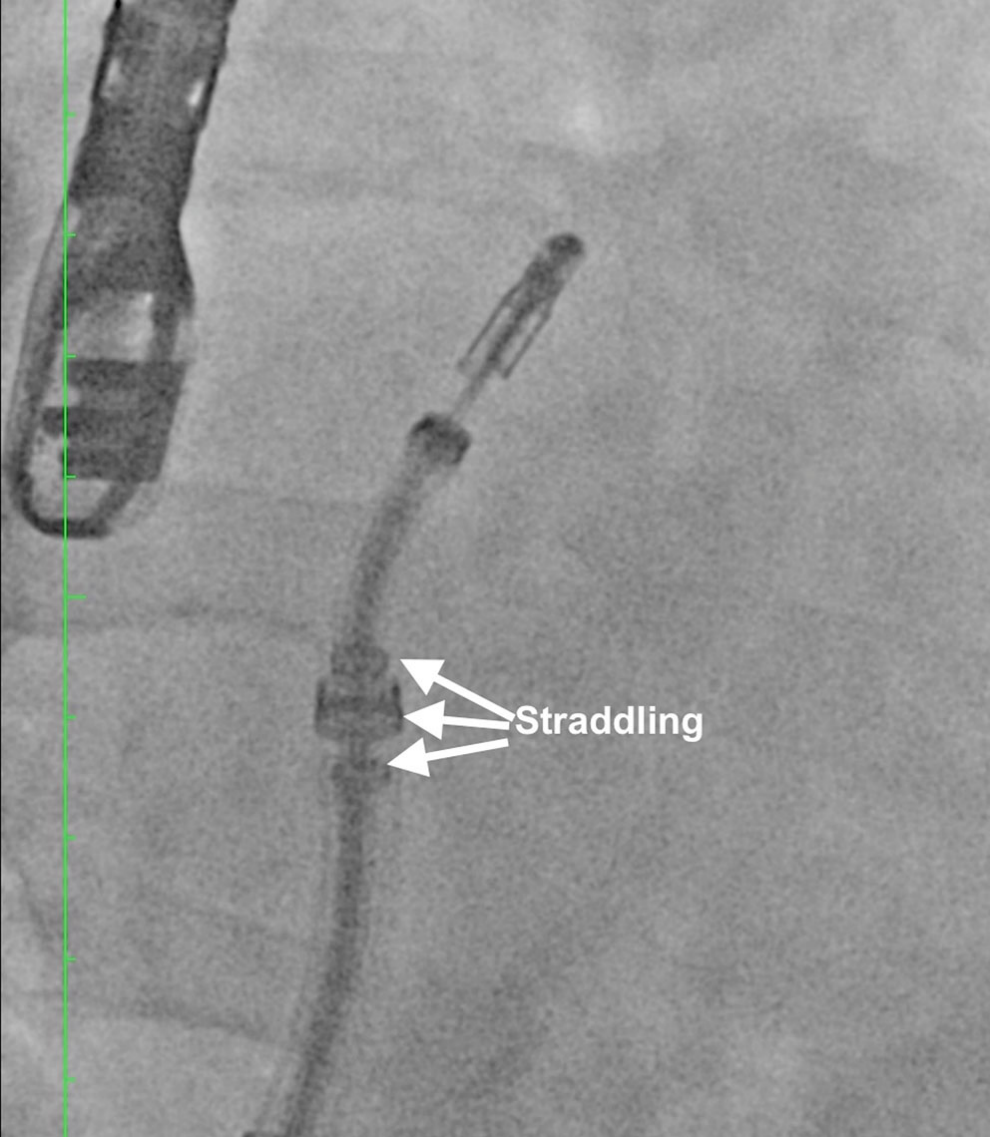
Steering and positioning of the MitraClip delivery system in the left atrium. The Clip Delivery System (CDS) is advanced until its tip is even with the guide tip under fluoroscopic guidance. The CDS is further advanced until the guide radiopaque tip ring marker is centred between the sleeve alignment markers with confirmation on fluoroscopy (straddling)

As the clip delivery system carefully exits the sheath it is required that the markers in the system straddle the sheath markers before the clip can be moved toward the mitral valve (Fig. 3).

To position the clip delivery system above the mitral valve, a posterior torque of the steerable guide catheter and simultaneous medial deflection of the clip delivery system accompanied by retraction of the whole system are required. These steps are done in our centre under complete fluoroscopic guidance. After bypassing the pulmonary ridge and the left atrial appendage, a gentle anterior rotation of the clip delivery system accompanied with a simple lateral rotation brings the clip above the mitral valve. The aim of this manoeuver is to direct the clip in a horizontal direction as seen in the postero-anterior view fluoroscopically.

**Fig. 4:**
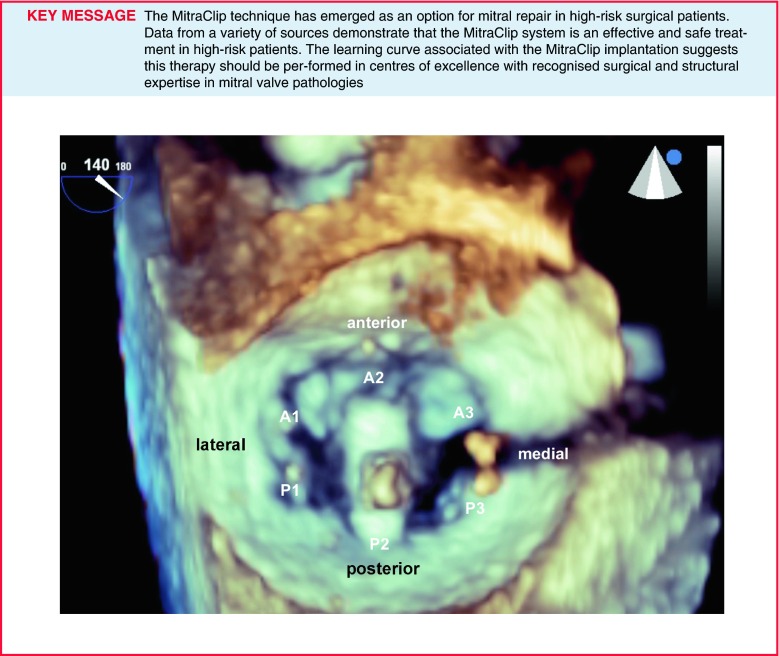
3D en face view allows to determine when the clip is adequately positioned above the middle segments of the mitral valve and whether orientation is perpendicular to the line of mitral valve coaptation

Medial-lateral clip adjustments are monitored in 3D en face view and anterior-posterior adjustments in an orthogonal mid-oesophageal long-axis (LVOT) view. To achieve good clip alignment, both arms of the opened clip can be visualised in full length in the long-axis view. The tip of the clip should be directed towards the largest proximal isovelocity surface area (PISA). A single 3D en face view allows to determine when the clip is adequately positioned above the middle segments of the mitral valve and whether orientation is perpendicular to the line of mitral valve coaptation (Fig. 4).

In the routine practice, it is recommended to pass the opened clip into the left ventricle [12]. In our experience, passing the mitral valve with an opened clip will require reassessment of the orientation of the clip again as the clip may rotate during translation from the left atrium to the left ventricle prolonging the procedure [13]. We prefer to advance the clip closed at the site of the regurgitant jet under the mitral leaflets in the LVOT view under breath-holding.

A correct orientation of the MitraClip, with perpendicular alignment to the line of coaptation, verifying that both mitral leaflets are freely moving above the clip arms, is crucial for a successful grasp of the mitral valve leaflets. To shorten the time of the procedure, we depend on the orientation of the clip above the mitral valve using 3D-TEE (Fig. 4) comparing it with the position of the clip fluoroscopically. Moreover, it is of outmost importance to find the corresponding view in 2D-TEE, where both arms of the clip are visible above the mitral valve. By doing this we define the correct angle in 2D-TEE in which we have to gasp the anterior and posterior leaflet in the PISA. This is the best and most simple way to ensure safe clipping with proper perpendicular alignment to the line of coaptation in the regurgitant area with no risk of valve distortion.

## Grasping of the leaflets, assessment of the result and clip release

Once the MitraClip is in a satisfactory position, grasping of the leaflets as they are captured in between the clip arms and the gripper is usually monitored using a 2D LVOT view. It is recommended to do this with the help of a short breath-hold, controlled by the accompanying anaesthetist. Multiple planes are useful for assessment of proper leaflet insertion into the MitraClip. The insertion of the posterior leaflet is commonly best seen in the LVOT view, and the insertion of the anterior leaflet in the four-chamber view. The intercommissural view can add information such as entrapped chordae tendinae.

**Fig. 5:**
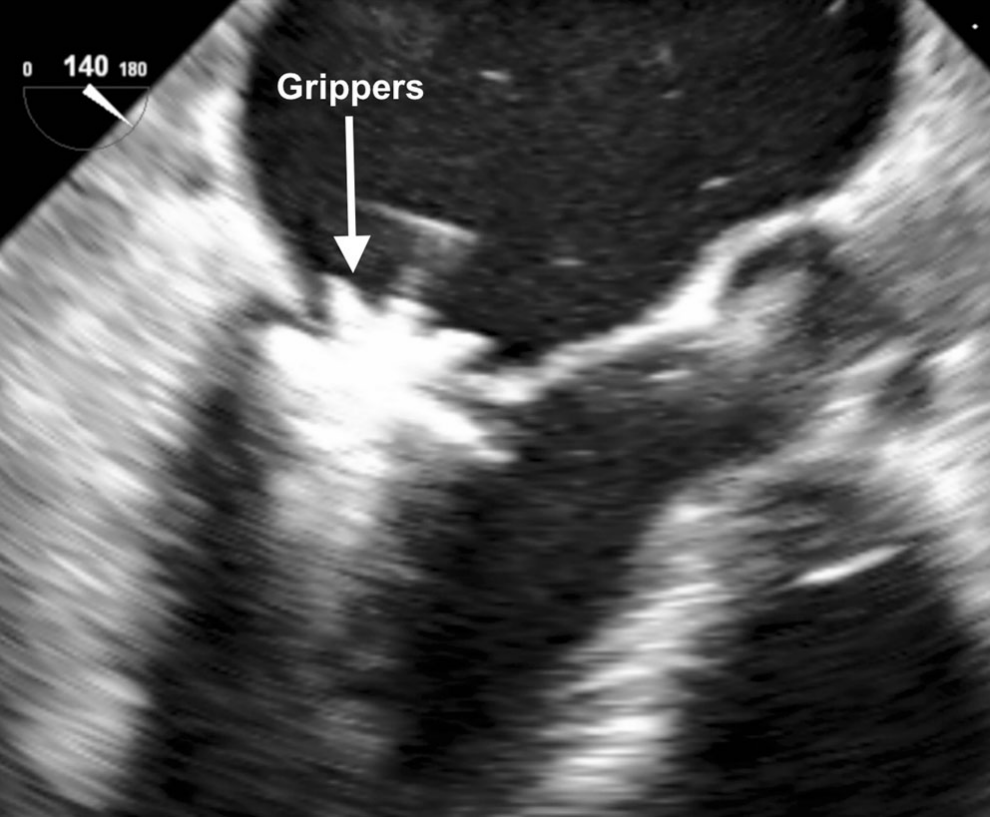
After grasping of the leaflets, leaflet capture is evaluated by lowering the gripper and partially closing the clip to secure leaflet insertion

After grasping of the leaflets, leaflet capture is evaluated by lowering the gripper and partially closing the clip to secure leaflet insertion (Fig. 5). Formation of the double-barrelled orifice is confirmed with an en face view to make sure that each orifice is approximately the same size.

Before and after the implantation of each clip, it is necessary to evaluate the grade of regurgitation and stenosis of the mitral valve, and in addition, the morphological result after clip placement [12].

Residual MR can easily be detected by colour Doppler. However, for quantification, it has to be taken into consideration that the area of colour jets is larger with multiple jets, which commonly occurs after a MitraClip is implanted (due to the addition of multiple jet areas), than if there is a single jet [14]. This may potentially lead to overestimating residual MR in patients with multiple jets. To overcome this problem, we recommend the measurement of the pressure curves in the left atrium to measure the height of the V‑wave before the procedure and compare it with the pressure curves after implantation of the MitraClip. This gives us an idea about the improvement of the degree of the MR. Left ventricular angiography may also help in evaluation the severity of the residual MR. On the other hand, angiographic MR grading may be inaccurate if the catheter is entrapped in the chordae tendineae, if insufficient contrast is used to opacify the left ventricle and left atrium, or in the presence of arrhythmias [15].

## Implantation of additional MitraClips

Our group has already proven that an optimal correction of MR after MitraClip placement should be advocated to optimise the benefits of the procedure and minimise the risk of adverse outcomes. As residual MR after MitraClip is associated with higher mortality than that for patients without MR [16].

The orientation of the second clip is mostly done fluoroscopically without the use of echocardiography and should be aligned as parallel as possible to the first clip.

During advancement of the MitraClip from the left atrium into the left ventricle under breath-holding, the clip should be closed to avoid any interference or entanglement with the chordae tendineae then re-opened in the left ventricle. Folding of leaflet tissue between two MitraClips should be avoided as this may cause uncorrectable residual MR [12].

## Conclusion

The MitraClip procedure is rapidly evolving as an important option among the current therapies for MR. The safety profile of this device appears to be good and data from experienced centres have shown that reduction of the severity of MR is achievable in almost all patients with low in-hospital complications and significant clinical improvement. Our simplified step-by-step approach helps us a lot.

Patients with moderate to severe or severe MR and suitable anatomical characteristics must be properly defined to ensure optimal clinical outcomes. In addition, TEE plays the major role in guidance of the procedure and in follow-up.

New technologies that synchronise echocardiographic and fluoroscopic imaging could potentially simplify and optimise procedural guidance, improve the degree of MR reduction, and enhance safety as well as shorten the duration of both fluoroscopy and the procedure time.

## References

[1] Glower DD, Kar S, Trento A (2014). Percutaneous mitral valve repair for mitral regurgitation in high-risk patients: results of the EVEREST II study. J Am Coll Cardiol.

[2] Feldman T, Wasserman HS, Herrmann HC (2005). Percutaneous mitral valve repair using the edge-to-edge technique: six-month results of the EVEREST Phase I Clinical Trial. J Am Coll Cardiol.

[3] Baldus S, Schillinger W, Franzen O (2012). MitraClip therapy in daily clinical practice: initial results from the German transcatheter mitral valve interventions (TRAMI) registry. Eur J Heart Fail.

[4] Schillinger W, Athanasiou T, Weicken N (2011). Impact of the learning curve on outcomes after percutaneous mitral valve repair with MitraClip and lessons learned after the first 75 consecutive patients. Eur J Heart Fail.

[5] Maisano F, Franzen O, Baldus S (2013). Percutaneous mitral valve interventions in the real world: early and 1‑year results from the ACCESS-EU, a prospective, multicenter, nonrandomized post-approval study of the MitraClip therapy in Europe. J Am Coll Cardiol.

[6] Grasso C, Capodanno D, Scandura S (2013). One- and twelve-month safety and efficacy outcomes of patients undergoing edge-to-edge percutaneous mitral valve repair (from the GRASP Registry). Am J Cardiol.

[7] Vakil K, Roukoz H, Sarraf M (2014). Safety and efficacy of the MitraClip(R) system for severe mitral regurgitation: a systematic review. Catheter Cardiovasc Interv.

[8] Feldman T, Kar S, Rinaldi M (2009). Percutaneous mitral repair with the MitraClip system: safety and midterm durability in the initial EVEREST (Endovascular Valve Edge-to-Edge REpair Study) cohort. J Am Coll Cardiol.

[9] Zamorano JL, Fernandez-Golfin C, Gonzalez-Gomez A (2015). Quantification of mitral regurgitation by echocardiography. Heart.

[10] Biner S, Perk G, Kar S (2011). Utility of combined two-dimensional and three-dimensional transesophageal imaging for catheter-based mitral valve clip repair of mitral regurgitation. J Am Soc Echocardiogr.

[11] Faletra FF, Pedrazzini G, Pasotti E, Moccetti T (2009). Real-time three-dimensional transoesophageal echocardiography showing sequential events of the percutaneous mitral clip procedure. Eur Heart J.

[12] Wunderlich NC, Siegel RJ (2013). Peri-interventional echo assessment for the MitraClip procedure. Eur Heart J Cardiovasc Imaging.

[13] Bhattacharya S, He Z (2015). Mechanics of mitral valve edge-to-edge-repair and MitraClip procedure. J Long Term Eff Med Implants.

[14] Quaife RA, Salcedo EE, Carroll JD (2014). Procedural guidance using advance imaging techniques for percutaneous edge-to-edge mitral valve repair. Curr Cardiol Rep.

[15] Grayburn PA, Weissman NJ, Zamorano JL (2012). Quantitation of mitral regurgitation. Circulation.

[16] Paranskaya L, D’Ancona G, Bozdag-Turan I (2013). Residual mitral valve regurgitation after percutaneous mitral valve repair with the MitraClip(R) system is a risk factor for adverse one-year outcome. Catheter Cardiovasc Interv.

